# The Role of Air Pollution in the Pathogenesis of Atopic Dermatitis, With a Focus on Oxidative Stress

**DOI:** 10.1002/clt2.70104

**Published:** 2025-09-15

**Authors:** Chen‐Xi Liu, Li Li, Yue‐Ping Zeng

**Affiliations:** ^1^ Department of Dermatology Plastic Surgery Hospital Chinese Academy of Medical Sciences and Peking Union Medical College Beijing China; ^2^ Department of Dermatology Peking Union Medical College Hospital Chinese Academy of Medical Sciences and Peking Union Medical College Beijing China

**Keywords:** air pollution, atopic dermatitis, immune response, inflammation, skin barrier

## Abstract

**Background:**

Atopic Dermatitis (AD) is a chronic inflammatory skin condition characterized by intensely itchy eczematous lesions and dryness. Recent epidemiological studies have indicated a notable increase in the prevalence of AD in industrialized countries, suggesting that air pollution may significantly influence the onset and progression of AD.

**Body:**

This review primarily describes the mechanistic roles of major air pollutants in the pathogenesis of AD, focusing particularly on oxidative stress, skin barrier dysfunction, and immune dysregulation. Moreover, the potential of targeting these pathways to prevent and manage AD is discussed.

**Conclusion:**

Air pollution contributes to the pathogenesis of AD by inducing oxidative stress, skin barrier dysfunction, and immune dysregulation through pathways such as AhR and NF‐κB. Mitigating its impact necessitates both personal protective measures and public health policies. Future research should investigate pollutant‐climate interactions and develop novel therapies targeting these mechanisms.

## 1 Background

**FIGURE 1:**
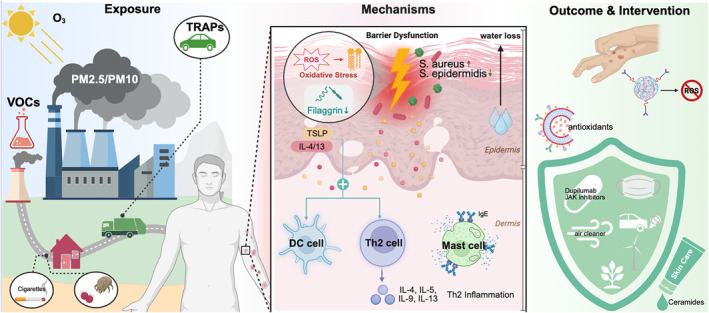
Proposed mechanisms of environmental pollutant‐induced skin damage and potential interventions.

Atopic Dermatitis (AD), a chronic inflammatory skin disease characterized by intense itch and barrier dysfunction, exhibits rising prevalence, particularly in industrialized regions [1]. Mounting epidemiological evidence strongly implicates air pollution as a significant environmental trigger and exacerbator of AD [2]. This review synthesizes current knowledge on the pathogenic role of air pollutants (e.g., PM, O_3_, NO_2_, VOCs), exploring how they damage to skin function and the immune system, and proposing other factors influencing the research such as climate and temperature. Based on the evaluation, we propose measures to cope with AD, including ideal lifestyles, future policies, and possible drug targets (Figure 1).

As the correlation between AD and air pollution has been established, more information is needed to further define the relationship. In this narrative review, we researched relevant evidence to evaluate the association between air pollution and AD objectively. The key words of “atopic dermatitis,” together with “air pollution,” “immune response,” “inflammation,” and diverse specific pollutants were searched in databases including PubMed, Web of Science, Embase, and Medline.

This schematic diagram systematically summarizes the pathogenic cascade through which environmental pollutants contribute to skin barrier disruption and immune dysregulation. Exposure to ozone (O_3_), nitrogen dioxide (NO_2_), particulate matter (PM_2.5_ and PM_10_), and volatile organic compounds (VOCs) induces oxidative stress and enhances reactive oxygen species (ROS) formation, leading to filaggrin downregulation and consequent impairment of the epidermal barrier. This dysfunction promotes microbial dysbiosis, characterized by increased colonization of *Staphylococcus aureus* and reduction of *Staphylococcus* epidermidis, which further activates dendritic cells, mast cells, and Th2 lymphocytes. These immune cells release cytokines such as IL‐4, IL‐13, and TSLP, driving Th2‐related immune responses and elevated IgE levels. Clinically, these mechanisms manifest as inflammatory skin conditions, which may be targeted through interventions including topical antioxidants, biologic therapies (e.g., dupilumab), JAK inhibitors, environmental controls such as air purification, and skin barrier repair agents featuring ceramides.

## 2 The Sources of Air Pollutants

Air pollutants are ubiquitous and can be categorized as outdoor and indoor pollutants. Outdoor pollutants have both natural and anthropogenic origins. Natural sources of pollution include wildfires, volcanic eruptions, dust storms, and biological decomposition processes. In contrast, anthropogenic emissions, which have escalated with industrialization and urbanization, arise from motor vehicles, power plants, manufacturing facilities, and incinerators. Indoor pollutants primarily originate from tobacco smoke, construction materials, and household products, which emit harmful gases. The United States Environmental Protection Agency establishes air quality standards for six air pollutants: particulate matter (PM), ground‐level ozone (O_3_), carbon monoxide (CO), sulfur dioxide (SO_2_), nitrogen dioxide (NO_2_), and lead [3]. Additional air pollutants include polycyclic aromatic hydrocarbons (PAH), volatile organic compounds (VOCs), other traffic‐related pollutants, and heavy metals such as nickel and cobalt. These air pollutants can significantly impact air quality in the residential environment, contributing to the onset and exacerbation of AD [4].

## 3 Epidemiology of Atopic Dermatitis

Atopic Dermatitis (AD) is a chronic inflammatory skin disease influenced by the interaction between multiple genetic and environmental factors. The overall prevalence of AD is approximately 20% in children and 10% in adults, varying across different countries and regions [5]. Recent studies have identified air pollution as a significant risk factor for the development of AD [6, 7]. A retrospective cohort study involving 209,168 Koreans demonstrated that long‐term exposure to air pollutants was significantly associated with the risk of AD in the general population. Specifically, a 1 μg/m^3^ increase in the long‐term average concentrations of PM_2.5_ and PM_10_ is correlated with increases in AD incidence of 42.0% and 33.3%, respectively. Additionally, SO_2_, NO_2_ and CO can also elevate the likelihood of developing AD [8].

Toluene diisocyanate can activate the TRPV1/TRPA1 calcium channels, stimulating nerve endings in the skin and inducing the release of neuropeptides (such as CCL27), leading to aggravated pruritus and prolonged cycles of itch and scratch [9, 10]. Long‐term exposure to pollutants can cause chronic inflammation and increase the frequency of AD recurrences. Furthermore, pollutants can elevate IgE levels and induce hypersensitivity to allergens [11]. Therefore, it is crucial to explore the effects of air pollution on the pathogenesis of AD and to develop targeted therapeutic strategies.

## 4 The Role of Air Pollutants in the Pathogenesis of Atopic Dermatitis

The key underlying mechanisms of AD include skin barrier dysfunction and immune dysregulation. Air pollutants (e.g., PM, O_3_, SO_2_) contribute to skin barrier damage by boosting oxidative stress and increasing transepidermal water loss. Additionally, these pollutants can trigger Th2‐mediated immune responses, resulting in heightened sensitization to allergens and elevated IgE levels, thereby inducing systemic allergic reactions [12]. Collectively, these factors—oxidative stress, skin barrier dysfunction, and immune response—contribute to an imbalance in the skin microbiome. It is important to note that the aforementioned mechanisms are not mutually exclusive; rather, they interact synergistically, ultimately leading to the onset or exacerbation of AD [13].

### 4.1 Skin Barrier Dysfunction

Air pollutants, including particulate matter (PM_2.5_ and PM_10_), ozone, sulfur dioxide, and nitrogen oxides stimulate the production of Reactive Oxygen Species (ROS), thereby mediating excessive oxidative stress in the skin. This oxidative stress damages DNA, lipids, and proteins, ultimately compromising the integrity of the skin barrier. Additionally, this process triggers inflammation and immune responses. The impaired integrity of the skin barrier facilitates the entry of pathogens and allergens, thereby exacerbating the inflammation associated with AD [14].

#### 4.1.1 Excess Oxidative Stress

Several studies have demonstrated that markers of oxidative stress are elevated while antioxidant levels are decreased in AD patients [15]. Ilves et al. conducted a study involving 15 AD patients and 17 controls, performing targeted metabolomic analysis of 188 metabolites from skin punch biopsies. Their findings indicated that notable alterations in the concentrations of amino acids, biogenic amines, and lipids are associated with oxidative stress, inflammation, and barrier function [16]. PM contains metal components and PAH, which can directly produce ROS through a Fenton‐type reaction and quinone redox cycling pathway [17]. Additionally, PM can stimulate ROS generation indirectly by activating aryl hydrocarbon receptor (AhR) or pattern recognition receptor (PRR) signaling pathways, such as Toll‐like receptors (TLRs) on keratinocytes [18]. Furthermore, PM disrupts mitochondrial function, leading to electron leakage from the electron transport chain and an increase in intracellular ROS levels [19]. A prospective study by Minzaghi et al. revealed that post‐endoplasmic reticulum stress induced by PM disrupts protein folding and triggers the unfolded protein response, resulting in ROS generation [20].

Excessive ROS combines with antioxidants, such as superoxide dismutase, depleting the antioxidant capacity and oxidative defense mechanisms of the skin. The imbalance between oxidants and antioxidants leads to oxidative stress, damaging DNA and proteins, and resulting in oxidative damage to keratinocytes [21]. The consequent reduction in intercellular adhesion and impairment of barrier function increases permeability and sensitivity to airborne pollutants and allergens, making the skin more vulnerable and prompting skin inflammation [22]. Pollutant‐induced cellular dysfunction disrupts the autophagy process, impeding the timely clearance of damaged proteins and mitochondria, and contributing to ROS accumulation and oxidative stress.

Ceramides constitute a significant portion of skin lipids, accounting for 40%–50% of the total lipids in the stratum corneum. Their production relies on the normal activity of various enzymes, including sphingomyelinase and sphingosine kinase [23]. Ceramides are significantly derived from the skin microbiome. For example, the abundant skin commensal *Staphylococcus* epidermidis secretes a sphingomyelinase that facilitates host production of ceramides to help maintain skin integrity and prevent water loss of damaged skin [24]. ROS can enhance lipid peroxidation, compromising the redox equilibrium of fatty acids. This leads to an increase in some saturated fatty acids or medium‐ and short‐chain fatty acids, while the content of long‐chain unsaturated fatty acids, which are crucial for maintaining skin hydration and flexibility, decreases. Such changes negatively affect skin softness and barrier stability. An early study indicated that exposure to O_3_ caused lipid peroxidation in the skin tissue, leading to the formation of malondialdehyde, a lipid oxidation byproduct, and oxidized proteins in the epidermis. Furthermore, short‐term exposure to O_3_ depletes levels of antioxidant vitamin C, uric acid and glutathione in the stratum corneum of mice, inducing oxidative stress responses and damaging the skin barrier [25]. ROS may also reduce the activity of lipid processing enzymes, decreasing the lipid content of the skin, particularly ceramide, and altering the intercellular lipid composition of the stratum corneum, which leads to higher transepidermal water loss (TEWL). This further weakens the skin barrier function, resulting in dry and pruritic skin. A reduced activity of paraoxonase, an important lipoprotein regulatory enzyme has also been observed in AD patients, potentially contributing to lipoprotein dysfunction [26].

Additionally, ROS can oxidize amino acid residues in proteins, forming carbonyl groups and disulfide bonds. This oxidative modification can affect the structure and function of proteins, impair cellular function, reduce enzyme activity, and disrupt receptor signaling. It damages the extracellular matrix and cytoskeleton in the skin, compromising the overall skin structure and barrier function [27].

#### 4.1.2 Abnormal Cuticle‐Related Structural Proteins

Filaggrin is a crucial component of stratum corneum structure, with its decomposition product functioning as a natural moisturizing factor that plays an essential role in epidermal hydration, lipid processing, and barrier function. Tight junctions are proteins that contribute to intercellular adhesion and the permeability barrier between cells. Pollutants can damage or inhibit tight junction proteins (such as claudin‐1 and occludin) and filaggrin in keratinocytes, directly impairing the skin barrier function [28]. For instance, PM can inhibit the gene expression of filaggrin, loricrin, keratin‐1, desmocollin‐1, and corneodesmosin, resulting in compromised skin barrier function [29]. Lecas et al. demonstrated that a single exposure to tobacco smoke altered the molecular composition of epidermal tissue, reducing loricrin staining in the epidermis, and stimulating the production of inflammatory cytokines (IL‐8, IL‐1α, and IL‐18) and matrix metalloproteinases (MMP‐1 and MMP‐3) [30]. Woo et al. found that exposure of mouse models to PM with a diameter of 10 μm or less damaged skin barrier integrity, promoted differential expression of immune response‐related genes, and induced or exacerbated AD [31]. Bae et al. used mouse models of oxazolone‐induced AD‐like skin and human keratinocytes. They showed that PM down‐regulates the expression of several cuticle and tight junction proteins, disrupting skin barrier function [32].

At the molecular level, oxidative‐antioxidative regulation in humans involves the alteration of signaling systems and gene expression. Dysregulation of ROS levels activates a series of signaling pathways, including mitogen‐activated protein kinase, Janus kinase (JAK) signal transducer and activator of transcription, phosphatidylinositol‐3‐kinase, and inflammatory mediators (including cytokines and chemokines). This dysregulation also affects the gene expression of related proteins, further compromising the skin barrier and fueling the inflammatory response [33].

### 4.2 Environmental Pollution Induces the Immune Imbalance and Inflammatory Responses

#### 4.2.1 Activation of Th2‐Type Immune Response

Air pollutants impair keratinocytes, stimulating them to secrete damage‐associated molecular patterns, thymic stromal lymphopoietin (TSLP), and cytokines such as IL‐4, IL‐13, and IL‐31, which can activate and recruit dendritic cells in the local microenvironment. After ingesting and processing pollutants or microbial‐derived antigens, DCs migrate to regional lymph nodes where they interact with T cells, leading to dysregulation of Th2‐related immune responses. This dysregulation further affects B cell differentiation and induces the production of IgE and eosinophils [34]. A classic experiment has demonstrated that pyrene in diesel exhaust particles can enhance IgE‐mediated allergic reactions and stimulate inflammatory responses in mice [35, 36]. Recently, Smith et al. identified a significant association between AD and e‐cigarette use among American adults, indicating that e‐cigarettes can drive Th2‐type immune responses and cytotoxic damage [37]. Kwack et al. employed dinitrochlorobenzene (DNCB) to induce AD‐like symptoms in mice. They showed that PM_10_ could upregulate the expression of inflammatory cytokines and TSLP, thereby increasing the serum IgE levels of mice [38]. A prospective study conducted by Tang et al. found that treatment of mouse bone marrow‐derived mast cells with VOCs (1,3‐butadiene and toluene), resulted in mast cell degranulation, releasing histamine, inducing inflammatory and allergic reactions [39]. Furthermore, long‐term exposure to pollutants can inhibit the immunomodulatory function of Treg cells, leading to immune dysregulation and chronic inflammation [40]. Treg cells (primarily Foxp3+ Treg) play a crucial role in inhibiting excessive inflammation and autoimmune responses. They secrete inhibitory cytokines (IL‐10 and TGF‐β) through cell‐contact‐dependent mechanisms to modulate immune response intensity. In some AD patients, the number of Treg has a dramatic reduction, accompanied by diminished function, which contributes to Th2‐type immune responses.

#### 4.2.2 Activation of Signaling Pathways Promotes the Release of Inflammatory Factors

The aryl hydrocarbon receptor (AhR) is a ligand‐dependent transcription factor generally expressed in all types of skin cells, playing a crucial role in epidermal differentiation, barrier function, and the maintenance of skin homeostasis [41]. Polycyclic aromatic hydrocarbons (PAH), significant organic pollutants found in PM and tobacco smoke, can bind to and continuously activate the AhR, forming the PAH‐AHR complex that translocates to the nucleus via a canonical pathway. Within the nucleus, AhR can dimerize with the AhR nuclear transporter to promote the transcription of target genes and upregulate the expression of proteins, including cytochrome P4501A1(CYP1A1), AKR1C3 and PDG2. This activation stimulates the production of inflammatory factors such as IL‐6 and IL‐13, while inhibiting the expression of filaggrin. Additionally, AhR promotes the migration of Langerhans cells [42, 43].

NF‐κB is a key regulator of pro‐inflammatory signaling pathways and integral to the production of various cytokines. Air pollutants modify the inhibitory protein IκBα through oxidative modification, allowing NF‐κB to translocate from the cytoplasm to the nucleus, where it binds to the promoter region of inflammatory genes. This process increases the expression of pro‐inflammatory factors such as IL‐1β, IL‐6, IL‐18, and TNF‐α, which further exacerbate skin inflammation by activating the NLRP3 inflammasome [44]. Interactions between pollutants and TLRs signaling also contribute to the activation of NF‐κB. Hergesell et al. observed the excessive production of ROS and the pro‐inflammatory factor IL‐6 after exposing the stratum corneum of porcine skin explants to cigarette smoke [45]. Piao et al. found that PM_2.5_ activates the NF‐κB pathway in keratinocytes, inducing the upregulation of inflammatory factors [46]. These inflammatory responses may also contribute to pathological conditions such as mitochondrial damage and autophagy disruption, ultimately leading to cell damage and apoptosis in skin cells [47]. McKee et al. suggested that metals can stimulate the PRR signaling pathway by directly activating PRRs, inducing cellular stress and resulting in cell death. Direct activation of PRR occurs through the binding of metal ions (such as nickel, cobalt, and palladium ions) to histidine residues (such as H456 and H458) on TLR4. This binding facilitates NF‐κB activation and the release of TNF‐α and IL‐8. Cellular oxidative stress following exposure to metal ions or particles contributes to lysosomal damage, activates the NLRP3 inflammasome, and ultimately leads to IL‐1β secretion [48].

### 4.3 Effects of Airborne Pollution on Skin Microbiome

Urbanization is linked to the diversity of skin microbiota, suggesting that air pollution may significantly impact the skin microbiome [49]. NO_2_ from traffic‐related air pollution (TRAP) has been shown to alter the composition of skin microbiota by disrupting the skin's pH and moisture levels [50]. Janvier et al. demonstrated NO_2_‐induced dysbiosis at 10–80 ppm—concentrations 200–1600‐fold higher than the WHO annual limit (0.05 ppm). Although such experimental doses reveal mechanistic hazards, their direct translation to environmental risks requires caution [51]. Nevertheless, these findings remain mechanistically relevant, as they provide insights into pollutant‐induced dysbiosis and cutaneous immune responses. Chronic low‐dose NO_2_ exposure (0.02–0.1 ppm) may still impair barrier function through cumulative effects or synergy with co‐pollutants, though population‐level risks are quantitatively distinct from high‐dose experimental outcomes. Furthermore, O_3_ can increase the colonization of pathogenic bacteria such as *Staphylococcus aureus*, while decreasing the abundance of normal skin flora, such as *Staphylococcus epidermidis*, thus compromising the skin barrier and enhancing the inflammatory response. Indoor pollutants, such as xylene and diisocyanates, also contribute to the imbalance of skin microbial flora and are associated with increased rates of AD in children [52]. Glyoxal present in PM promotes *Staphylococcus aureus* colonization and exacerbates symptoms in rat models of AD [53]. Additionally, dysregulated skin microbiota further intensifies, in turn, exacerbates immune dysregulation and epidermal barrier disruption in AD patients by impairing filaggrin function, disrupting tight junctions, and modulating immune response genes. For example, *S. aureus* indirectly inhibits the expression of fatty acid elongation enzymes (ELOVL3 and ELOVL4) in HEK via IL‐1β, TNF‐α, IL‐6, and IL‐33, leading to increased TEWL in organotypic skin [54]. Furthermore, virulence factors such as enterotoxins and proteases secreted by *S. aureus* strongly stimulate inflammation and disrupt local immune regulation, continuously amplifying ROS production and activating pro‐inflammatory pathways.

### 4.4 Epigenetic Alterations

Recently, the incidence of allergic diseases, which was thought to be associated only with genetic predisposition and environmental triggers, is increasingly linked to the esoteric but significant field of epigenetic modifications. These modifications—including DNA methylation, histone modifications, and alterations in microRNA (miRNA) expression—act as molecular “switches” that occur and regulate gene expression under the influence of allergens and air pollutants [55]. Additionally, epigenetic features have the potential for transgenerational transmission; prior exposure to pollutants can increase the risk of allergic diseases in offspring. For example, Sun et al. highlighted that PM_2.5_ enhanced the expression of glutamate oxaloacetate transaminase 1 through AhR and its promoter, resulting in the hypermethylation of the FOXP3 locus and impairing the differentiation of Treg cells [56]. Rashmi et al. found that tobacco smoke could increase the expression of miRNA‐223 in maternal blood and reduce the number of Treg cells in the umbilical cord blood of infants observed DNA methylation [57]. Jung et al. concluded that exposure to air pollution during pregnancy significantly affected cytokine and histone modification profiles at single‐cell levels [58]. Similarly, fetal exposure to air pollution has been implicated in causing pro‐inflammatory innate and adaptive immune effects as well as epigenetic changes. Furthermore, it has been demonstrated that miRNA‐146a‐5p is crucial in regulating immune responses and is associated with IgE levels in AD [59]. Liu et al.'s study in mice showed that exposure to diesel exhaust particulates increased IgE production after exposure to the allergen (Aspergillus fumigatus) through hypermethylation of IFN‐γ and hypomethylation of IL4 [60]. It is through these intricate processes that epigenetic modifications not only act as “bystanders” of immunological changes, but also influence the direction of immune fate to a certain extent.

## 5 Limitations of Current Research and Expected Research Directions in the Future

### 5.1 Synergies Between Exposome and Air Pollution

Besides urbanization and air pollution, climatic factors such as seasonality, temperature, humidity, and ultraviolet rays interact with air pollutants, and fluctuations in these parameters may mitigate or exacerbate the impact of air pollutants on AD [61]. As we all know, global warming is currently the biggest climate problem, and studies have shown that climate warming is related to the pathogenesis of AD [62]. Guo et al. found that the link between air pollutants and hospital outpatient visits of AD may also be attributed to meteorological factors, particularly high temperatures [63]. High temperatures can promote sweat secretion, and increased concentrations of salt and lactate in sweat can weaken the lipid structure of the stratum corneum, exacerbating skin dryness and itching. The increase in temperature due to global warming can lead to the destruction of the skin microbiota, which can exacerbate AD [64]. In addition, since atmospheric humidity also interacts with pollutants, the study investigated the correlation between humidity and different levels of air pollutants, as well as the relationship between humidity and AD. PM_2.5_ and PM_10_ levels were negatively correlated with humidity. Abrupt changes from a high‐humidity environment to a low‐humidity environment can lead to abnormal barrier function, accelerating the TEWL of the skin of AD patients, thereby amplifying barrier defects and increasing cytokine signaling to promote inflammatory responses [65]. Kim et al. showed that the risk of AD symptoms due to PM_2.5_ and PM_10_ exposure was significantly increased during periods of moderate dryness [66]. Additionally, the combination of UVA with ozone can cause synergistic oxidative stress in human skin [67, 68]. Although it is known that climate factors affect air quality, further research is needed on how the interaction between the two exacerbates or mitigates the skin effects of air pollutants [69].

### 5.2 Population Susceptibility

The potential health risks posed by air pollution not only depend on the level of exposure or composition of the pollutant, but also on the host's susceptibility, such as higher incidence rates for children, mothers and people with genetic susceptibility. A study in Shanghai found that air pollution increases the number of pediatric AD clinics, especially children under the age of 6 are more susceptible to its impact [70]. This may be related to children's thinner skin and weaker barrier function. Furthermore, prenatal environmental exposure in pregnant women is related to the incidence of AD. Many studies have shown that prenatal exposure to air pollutants such as PM_2.5_, NO_2_, heavy metals (nickel and lead), cigarette smoke, etc. may increase the risk of AD in children and have varying degrees of impact at different stages of pregnancy [71, 72].

### 5.3 Metabolomics

When air pollution is severe, outdoor activities decrease, resulting in increased exposure to indoor allergens and may also aggravate AD. Common allergens indoors include bioaerosols (mold or bacteria), house dust mite and dust. A South Korean study found that elevated indoor humidity increased mold and bacterial content, correlating with higher AD incidence in children with prolonged exposure [73]. Another study showed that prenatal exposure to mold is associated with AD, that is, infants with AD exposed to mold during pregnancy had higher total serum IgE levels at 1 year old than healthy infants who were not exposed to mold during pregnancy (*p* = 0.021) [74].

It is notable that pollutants in the air exist in the form of a mixture of multiple harmful gases, and most current studies are limited to exposure conditions to a single pollutant. There are few studies on co‐exposed multiple pollutants to promote inflammatory response. Metabolomics analysis may make up for this shortcoming [75]. With the development of material technology, new materials may also become air pollutants. For example, nano‐scale particulate matter (such as titanium dioxide and carbon black) can penetrate the skin barrier, inducing local inflammatory responses and oxidative stress, while microplastics may indirectly affect the pathogenesis of AD by adsorbing other harmful substances, such as heavy metals and polycyclic aromatic hydrocarbons [76, 77, 78].

New data show that neonatal ceramide, phospholipid and sphingolipid levels may evolve into the next‐generation biomarker for AD [79, 80]. Therefore, future lipomics studies on the onset of AD may be more accurate and reliable [81].

### 5.4 Interactions Between Environmental, Genetic, and Immune Systems

To better understand the multifactorial nature of AD, it is important to study the relationship between environmental, genetic, and immune factors. Gene‐environment interaction studies have highlighted the important association between AD and air pollution. Specifically, genes belonging to the Glutathione S‐transferase (GST) family are of particular interest because of their role in cellular protection against oxidative stress [82]. Evidence has found that children with GST pi 1 (*GSTP1*) and GST Mu 1 (*GSTM1*) genotypes may constitute a susceptible population at increased risk of childhood AD associated with prenatal smoke exposure [83]. However, this observation represents an association, not causation. The study did not exclude the possibility that shared familial behaviors—such as smoking, which influences mate selection—may confound the relationship. Therefore, GST polymorphisms may act as markers of gene‐environment interaction rather than direct causal agents. Notably, even in the presence of genetic susceptibility, the development of AD still depends on actual environmental exposure to relevant pollutants. In addition, people with mutations in the *FLG* or *AKR1C3* genes were associated with more significant skin barrier function impairment and inflammatory responses when exposed to pollutants [84, 85]. However, there are also patients with normal *FLG* genes in AD patients. An example of a genetic‐environmental interaction is the observation that endotoxin exposure reduces the risk of sensitization in subjects with a specific phenotype of lipopolysaccharide receptor CD14 encoded by chromosome 5q31.1 [86, 87]. More studies are needed to elucidate the interactions between these three and their impact on the pathogenesis of AD. Due to the spatiotemporal dynamics of epigenetics and the complexity of the interaction mechanism between environment and genetics, future research may need to combine single‐cell sequencing or multi‐omics integration models (epigenome + genome + environmental exposome). Epigenetics provides a new perspective on the pathogenesis of AD, revealing how environmental factors affect the disease process by dynamically regulating gene expression. Future research needs to further identify key epigenetic markers, develop targeted therapy strategies, and promote clinical translation through interdisciplinary collaboration, so as to achieve precise prevention and management of AD.

## 6 Targeted Measures to Prevent and Control AD Induced by Air Pollutants

To mitigate the adverse effects of air pollution, individuals should minimize direct exposure to pollutants through the selection of non‐irritating materials and outdoor mask wearing, limit outdoor activities in areas with high levels of PM, VOCs, and TRAP. Basic emollients can help restore the skin barrier function and may assist in improving AD control by reducing sensitivity to irritants and allergens which contains urea, glycerol, petrolatum, vitamin E, and vegetable oils [88]. Furthermore, the skin barrier function may be enhanced through the application of skincare products with ceramides [89, 90]. A novel zinc lactobionate emollient cream can strengthen the skin barrier by lowering skin surface pH in patients with atopic dermatitis [91]. Notably, anti‐inflammatory drugs targeting particulate matter are under investigation, including rosmarinic acid, camellia extract, and coffee cherry pulp, which may potentially serve as ingredients in skin care products [92, 93, 94]. Recent research has identified tapinarof, a novel small‐molecule topical aryl hydrocarbon receptor agonist, which has demonstrated efficacy and safety in phase III clinical trials [95]. For managing Th2‐type inflammatory responses triggered by pollutants, treatment options include topical anti‐inflammatory drugs (such as glucocorticoids or tacrolimus), biologic agents (such as dupilumab, an anti‐IL‐4Rα monoclonal antibody), and small molecule inhibitors (such as JAK1/2 inhibitors) [96]. In addition, innovative treatments and topical drug delivery systems have become hot spots for future research. For example, how nanomaterials can be used to encapsulate antioxidants and other drugs to improve their bioavailability and therapeutic efficacy [97]. Semi‐permeable “second skin” films containing active substances such as enzymes or metal‐organic frameworks can also be prepared to capture and break down pollutants upon exposure to pollutants such as PM_2.5_ and VOCs while maintaining the skin's normal breathability [98]. In addition, the use of enzyme‐like catalysts (nanozymes) to stabilize the distribution on the surface of the skin to continuously scavenge ROS and neutralize free radicals generated by exposure to pollutants can also be considered [99]. Successful urban planning can help reduce air pollution by optimizing city design with regard to transportation systems. For instance, urban planning can reduce air pollution by enhancing the use of renewable energy, increasing green space and choosing alternative transport systems [100]. Women and children are most frequently exposed to high levels of indoor air pollutants, so improving home air ventilation by installing chimneys, air filtration devices and modern stoves that use safer fuels can reduce the health burden of indoor pollution [101, 102]. Furthermore, the government should formulate emission reduction policies to promote the development of a low‐carbon economy [103, 104]. These large‐scale adjustments will require combined efforts of the healthcare, public health, construction development and mechanical engineering industries. As these adjustments require significant time for implementation, additional research on methods of reversing pollutant‐induced skin barrier damage will be critical in the coming years.

## 7 Conclusion

Air pollutants contribute to the pathogenesis of AD by damaging the skin barrier, inducing oxidative stress, stimulating Th2‐type immune responses [105], promoting the release of pro‐inflammatory factors, and altering the microbiota [106]. Enhancing the living environment and implementing proactive skincare measures are essential for improving human living standards and global health quality.

## Author Contributions

**Chen‐Xi Liu:** writing – original draft, writing – review and editing. **Li Li:** writing – review and editing. **Yue‐Ping Zeng:** writing – review and editing.

## Conflicts of Interest

The authors declare no conflicts of interest.

## Data Availability

Data sharing not applicable to this article as no datasets were generated or analyzed during the current study.

## References

[1] Y. Ai , J. Huang , and T. T. Zhu , “Early Exposure to Maternal Stress and Risk for Atopic Dermatitis in Children: A Systematic Review and Meta‐Analysis,” Clinical and Translational Allergy 14, no. 3 (2024): e12346, 10.1002/clt2.12346. 38488856 PMC10941798

[2] H. Y. Huang , K. H. Sheen , C. Y. Hung , et al., “Association Between Telomere Length and Atopic Dermatitis Among School‐Age Children,” Clinical and Translational Allergy 15, no. 6 (2025): e70066, 10.1002/clt2.70066. 40437344 PMC12119238

[3] U.S. Environmental Protection Agency . Criteria Air Pollutants (U.S. Environmental Protection Agency, 2014), https://www.epa.gov/criteria‐air‐pollutants/naaqs‐table.

[4] C.‐Y. Wu , C.‐Y. Wu , M.‐C. Li , H. J. Ho , and C.‐K. Ao , “Association of Air Quality Index (AQI) With Incidence of Atopic Dermatitis in Taiwan: A Nationwide Population‐Based Cohort Study,” Journal of the American Academy of Dermatology 90, no. 6 (2024): 1218–1225, 10.1016/j.jaad.2024.01.058. 38311242

[5] A. Mosam and G. Todd , “Global Epidemiology and Disparities in Atopic Dermatitis,” British Journal of Dermatology 188, no. 6 (2023): 726–737, 10.1093/bjd/ljad042. 36881991

[6] H.‐J. Jeon , H.‐J. Jeon , and S. H. Jeon , “Predicting the Daily Number of Patients for Allergic Diseases Using PM10 Concentration Based on Spatiotemporal Graph Convolutional Networks,” PLoS One 19, no. 6 (2024): e0304106, 10.1371/journal.pone.0304106. 38870112 PMC11175429

[7] K. P. Santiago Mangual , S. Ferree , J. E. Murase , and A. S. Kourosh , “The Burden of Air Pollution on Skin Health: A Brief Report and Call to Action,” Dermatologic Therapy 14, no. 1 (2024): 251–259, 10.1007/s13555-023-01080-1. PMC10828340 38103119

[8] S. K. Park , J. S. Kim , and H.‐M. Seo , “Exposure to Air Pollution and Incidence of Atopic Dermatitis in the General Population: A National Population‐Based Retrospective Cohort Study,” Journal of the American Academy of Dermatology 87, no. 6 (2022): 1321–1327, 10.1016/j.jaad.2021.05.061. 34242692

[9] M. Yadav , P. P. Chaudhary , B. N. D’Souza , et al., “Diisocyanates Influence Models of Atopic Dermatitis Through Direct Activation of TRPA1,” PLoS One 18, no. 3 (2023): e0282569, 10.1371/journal.pone.0282569. 36877675 PMC9987805

[10] A.‐P. Koivisto , M. G. Belvisi , R. Gaudet , and A. Szallasi , “Advances in TRP Channel Drug Discovery: From Target Validation to Clinical Studies,” Nature Reviews Drug Discovery 21, no. 1 (2022): 41–59, 10.1038/s41573-021-00268-4. 34526696 PMC8442523

[11] X. Gu , Z. Li , and J. Su , “Air Pollution and Skin Diseases: A Comprehensive Evaluation of the Associated Mechanism,” Ecotoxicology and Environmental Safety 278 (2024): 116429, 10.1016/j.ecoenv.2024.116429. 38718731

[12] Z. Pan , Y. Dai , N. Akar‐Ghibril , et al., “Impact of Air Pollution on Atopic Dermatitis: A Comprehensive Review,” Clinical Reviews in Allergy and Immunology 65, no. 2 (2023): 121–135, 10.1007/s12016-022-08957-7. 36853525

[13] J. Zeldin , G. Ratley , N. Shobnam , and I. A. Myles , “The Clinical, Mechanistic, and Social Impacts of Air Pollution on Atopic Dermatitis,” Journal of Allergy and Clinical Immunology 154, no. 4 (2024): 861–873, 10.1016/j.jaci.2024.07.027. 39151477 PMC11456380

[14] B. E. Kim , J. W. Hui‐Beckman , M. Z. Nevid , E. Goleva , and D. Y. M. Leung , “Air Pollutants Contribute to Epithelial Barrier Dysfunction and Allergic Diseases,” Annals of Allergy, Asthma, & Immunology 132, no. 4 (2024): 433–439, 10.1016/j.anai.2023.11.014. 38006973

[15] A. A. Hebert , “Oxidative Stress as a Treatment Target in Atopic Dermatitis: The Role of Furfuryl Palmitate in Mild‐to‐Moderate Atopic Dermatitis,” International Journal of Women's Dermatology 6, no. 4 (2020): 331–333, 10.1016/j.ijwd.2020.03.042. PMC7522904 33015298

[16] L. Ilves , A. Ottas , B. Kaldvee , et al., “Metabolomic Analysis of Skin Biopsies From Patients With Atopic Dermatitis Reveals Hallmarks of Inflammation, Disrupted Barrier Function and Oxidative Stress,” Acta Dermato‐Venereologica 101, no. 2 (2021): adv00407, 10.2340/00015555-3766. 33585945 PMC9366688

[17] L. Zhang , Y. Jin , M. Huang , and T. M. Penning , “The Role of Human Aldo‐Keto Reductases in the Metabolic Activation and Detoxication of Polycyclic Aromatic Hydrocarbons: Interconversion of PAH Catechols and PAH o‐quinones,” Frontiers in Pharmacology 3 (2012 2012): 193, 10.3389/fphar.2012.00193. PMC3499756 23162467

[18] R. Fitoussi , M.‐O. Faure , G. Beauchef , and S. Achard , “Human Skin Responses to Environmental Pollutants: A Review of Current Scientific Models,” Environment and Pollution 306 (2022): 119316, 10.1016/j.envpol.2022.119316. 35469928

[19] L. Chao , B. Feng , H. Liang , X. Zhao , and J. Song , “Particulate Matter and Inflammatory Skin Diseases: From Epidemiological and Mechanistic Studies,” Science of the Total Environment 905 (2023): 167111, 10.1016/j.scitotenv.2023.167111. 37716690

[20] D. Minzaghi , P. Pavel , C. Kremslehner , et al., “Excessive Production of Hydrogen Peroxide in Mitochondria Contributes to Atopic Dermatitis,” Journal of Investigative Dermatology 143, no. 10 (2023): 1906–1918.e8, 10.1016/j.jid.2023.03.1680. 37085042

[21] I. M. Dijkhoff , B. Drasler , B. B. Karakocak , et al., “Impact of Airborne Particulate Matter on Skin: A Systematic Review From Epidemiology to In Vitro Studies,” Particle and Fibre Toxicology 17, no. 1 (2020): 35, 10.1186/s12989-020-00366-y. 32711561 PMC7382801

[22] C. Alessandrello , S. Sanfilippo , P. L. Minciullo , and S. Gangemi , “An Overview on Atopic Dermatitis, Oxidative Stress, and Psychological Stress: Possible Role of Nutraceuticals as an Additional Therapeutic Strategy,” International Journal of Molecular Sciences 25, no. 9 (2024): 5020, 10.3390/ijms25095020. 38732239 PMC11084351

[23] F. Akiyama , N. Takahashi , Y. Ueda , et al., “Correlations Between Skin Condition Parameters and Ceramide Profiles in the Stratum Corneum of Healthy Individuals,” International Journal of Molecular Sciences 25, no. 15 (2024): 8291, 10.3390/ijms25158291. 39125861 PMC11311646

[24] Y. Zheng , R. L. Hunt , A. E. Villaruz , et al., “Commensal Staphylococcus Epidermidis Contributes to Skin Barrier Homeostasis by Generating Protective Ceramides,” Cell Host & Microbe 30, no. 3 (2022): 301–313, 10.1016/j.chom.2022.01.004. 35123653 PMC8917079

[25] J. J. Thiele , M. G. Traber , T. G. Polefka , C. E. Cross , and L. Packer , “Ozone‐Exposure Depletes Vitamin E and Induces Lipid Peroxidation in Murine Stratum Corneum,” Journal of Investigative Dermatology 108, no. 5 (1997): 753–757, 10.1111/1523-1747.ep12292144. 9129228

[26] O. Simonetti , T. Bacchetti , G. Ferretti , et al., “Oxidative Stress and Alterations of Paraoxonases in Atopic Dermatitis,” Antioxidants 10, no. 5 (2021): 697, 10.3390/antiox10050697. 33925093 PMC8144960

[27] Y. Niwa , H. Sumi , K. Kawahira , T. Terashima , T. Nakamura , and H. Akamatsu , “Protein Oxidative Damage in the Stratum Corneum: Evidence for a Link Between Environmental Oxidants and the Changing Prevalence and Nature of Atopic Dermatitis in Japan,” British Journal of Dermatology 149, no. 2 (2003): 248–254, 10.1046/j.1365-2133.2003.05417.x. 12932228

[28] K. Paik , J.‐I. Na , C.‐H. Huh , and J.‐W. Shin , “Particulate Matter and Its Molecular Effects on Skin: Implications for Various Skin Diseases,” International Journal of Molecular Sciences 25, no. 18 (2024): 9888, 10.3390/ijms25189888. 39337376 PMC11432173

[29] B. E. Kim , J. Kim , E. Goleva , et al., “Particulate Matter Causes Skin Barrier Dysfunction,” JCI Insight 6, no. 5 (2021): e145185, 10.1172/jci.insight.145185. 33497363 PMC8021104

[30] S. Lecas , E. Boursier , R. Fitoussi , et al., “In Vitro Model Adapted to the Study of Skin Ageing Induced by Air Pollution,” Toxicology Letters 259 (2016): 60–68, 10.1016/j.toxlet.2016.07.026. 27480279

[31] Y. R. Woo , S.‐Y. Park , K. Choi , E. S. Hong , S. Kim , and H. S. Kim , “Air Pollution and Atopic Dermatitis (AD): The Impact of Particulate Matter (PM10) on an AD Mouse‐Model,” International Journal of Molecular Sciences 21, no. 17 (2020): 6079, 10.3390/ijms21176079. 32846909 PMC7503766

[32] Y. J. Bae , K. Y. Park , H. S. Han , et al., “Effects of Particulate Matter in a Mouse Model of Oxazolone‐Induced Atopic Dermatitis,” Annals of Dermatology 32, no. 6 (2020): 496–507, 10.5021/ad.2020.32.6.496. 33911793 PMC7875236

[33] M. Nie , A. L. Blankenship , and J. P. Giesy , “Interactions Between Aryl Hydrocarbon Receptor (Ahr) and Hypoxia Signaling Pathways,” Environmental Toxicology and Pharmacology 10, no. 1–2 (2001): 17–27, 10.1016/s1382-6689(01)00065-5. 11382553

[34] P. Karimi , K. O. Peters , K. Bidad , and P. T. Strickland , “Polycyclic Aromatic Hydrocarbons and Childhood Asthma,” European Journal of Epidemiology 30, no. 2 (2015): 91–101, 10.1007/s10654-015-9988-6. 25600297

[35] A. Tsien , D. Diaz‐Sanchez , J. Ma , and A. Saxon , “The Organic Component of Diesel Exhaust Particles and Phenanthrene, a Major Polyaromatic Hydrocarbon Constituent, Enhances Ige Production by IgE‐secreting EBV‐Transformed Human B Cells in Vitro,” Toxicology and Applied Pharmacology 142, no. 2 (1997): 256–263, 10.1006/taap.1996.8063. 9070347

[36] T. Kanoh , T. Suzuki , M. Ishimori , et al., “Adjuvant Activities of Pyrene, Anthracene, Fluoranthene and Benzo(A)Pyrene in Production of Anti‐Ige Antibody to Japanese Cedar Pollen Allergen in Mice,” Journal of Clinical & Laboratory Immunology 48, no. 4 (1996): 133–147, PMID: 9819666. 9819666

[37] B. Smith , P. Engel , M. R. Collier , et al., “Association Between Electronic‐Cigarette Use and Atopic Dermatitis Among United States Adults,” Journal of the American Academy of Dermatology 89, no. 1 (2023): 163–165, 10.1016/j.jaad.2023.02.027. 36842506

[38] M. H. Kwack , J. S. Bang , and W. J. Lee , “Preventative Effects of Antioxidants Against PM10 on Serum Ige Concentration, Mast Cell Counts, Inflammatory Cytokines, and Keratinocyte Differentiation Markers in DNCB‐Induced Atopic Dermatitis Mouse Model,” Antioxidants 11, no. 7 (2022): 1334, 10.3390/antiox11071334. 35883825 PMC9311925

[39] K.‐T. Tang , Y.‐S. Chen , M.‐F. Lee , et al., “Exposure to Volatile Organic Compounds May Contribute to Atopic Dermatitis in Adults,” Biomedicines 12, no. 7 (2024): 1419, 10.3390/biomedicines12071419. 39061993 PMC11274632

[40] R. Raqib , E. Akhtar , H. M. Ahsanul , et al., “Reduction of Household Air Pollution Through Clean Fuel Intervention and Recovery of Cellular Immune Balance,” Environment International 179 (2023): 108137, 10.1016/j.envint.2023.108137. 37579572 PMC11062205

[41] A. Wazir and E. A. O'Toole , “Itching for Innovation: Role of Aryl Hydrocarbon Receptor Agonists as a Future Therapy for Atopic Dermatitis,” Clinical and Experimental Dermatology (2024): llae502, 10.1093/ced/llae502. 39570674

[42] H. Jin , Z. Lin , T. Pang , et al., “Effects and Mechanisms of Polycyclic Aromatic Hydrocarbons in Inflammatory Skin Diseases,” Science of the Total Environment 925 (2024): 171492, 10.1016/j.scitotenv.2024.171492. 38458465

[43] S. P. Proper , A. T. Dwyer , A. Appiagyei , et al., “Aryl Hydrocarbon Receptor and IL‐13 Signaling Crosstalk in Human Keratinocytes and Atopic Dermatitis,” Front Allergy 5 (2024): 1323405, 10.3389/falgy.2024.1323405. 38344408 PMC10853333

[44] F. Ferrara , R. Prieux , B. Woodby , and G. Valacchi , “Inflammasome Activation in Pollution‐Induced Skin Conditions,” supplement, Plastic and Reconstructive Surgery 147, no. 1S–2 (2021): 15S–24S, 10.1097/PRS.0000000000007617. 33347070

[45] K. Hergesell , K. Valentová , V. Velebný , K. Vávrová , and I. Dolečková , “Common Cosmetic Compounds Can Reduce Air Pollution‐Induced Oxidative Stress and Pro‐Inflammatory Response in the Skin,” Skin Pharmacology and Physiology 35, no. 3 (2022): 156–165, 10.1159/000522276. 35100602

[46] M. J. Piao , M. J. Ahn , K. A. Kang , et al., “Particulate Matter 2.5 Damages Skin Cells by Inducing Oxidative Stress, Subcellular Organelle Dysfunction, and Apoptosis,” Archives of Toxicology 92, no. 6 (2018): 2077–2091, 10.1007/s00204-018-2197-9. 29582092 PMC6002468

[47] F. Cervellati , M. Benedusi , F. Manarini , et al., “Proinflammatory Properties and Oxidative Effects of Atmospheric Particle Components in Human Keratinocytes,” Chemosphere 240 (2020): 124746, 10.1016/j.chemosphere.2019.124746. 31568946

[48] A. S. McKee and A. P. Fontenot , “Interplay of Innate and Adaptive Immunity in Metal‐Induced Hypersensitivity,” Current Opinion in Immunology 42 (2016): 25–30, 10.1016/j.coi.2016.05.001. 27228132 PMC5086267

[49] L.‐I. McCall , C. Callewaert , Q. Zhu , et al., “Home Chemical and Microbial Transitions Across Urbanization,” Nature Microbiology 5, no. 1 (2020): 108–115, 10.1038/s41564-019-0593-4. PMC7895447 31686026

[50] M. H. Y. Leung , X. Tong , Z. Shen , et al., “Skin Microbiome Differentiates Into Distinct Cutotypes With Unique Metabolic Functions Upon Exposure to Polycyclic Aromatic Hydrocarbons,” Microbiome 11, no. 1 (2023): 124, 10.1186/s40168-023-01564-4. 37264459 PMC10233911

[51] X. Janvier , S. Alexandre , A. M. Boukerb , et al., “Deleterious Effects of an Air Pollutant (NO2) on a Selection of Commensal Skin Bacterial Strains, Potential Contributor to Dysbiosis?,” Frontiers in Microbiology 11 (2020): 591839, 10.3389/fmicb.2020.591839. 33363523 PMC7752777

[52] G. Ratley , J. Zeldin , A. A. Sun , M. Yadav , P. P. Chaudhary , and I. A. Myles , “Spatial Modeling Connecting Childhood Atopic Dermatitis Prevalence With Household Exposure to Pollutants,” Communication and Medicine 4, no. 1 (2024): 74, 10.1038/s43856-024-00500-3. PMC11026442 38637696

[53] R. T. Han , H. Y. Kim , H. Ryu , et al., “Glyoxal‐Induced Exacerbation of Pruritus and Dermatitis Is Associated With Staphylococcus Aureus Colonization in the Skin of a Rat Model of Atopic Dermatitis,” Journal of Dermatological Science 90, no. 3 (2018): 276–283, 10.1016/j.jdermsci.2018.02.012. 29496360

[54] J. Kim , B. E. Kim , E. Berdyshev , et al., “Staphylococcus Aureus Causes Aberrant Epidermal Lipid Composition and Skin Barrier Dysfunction,” Allergy 78, no. 5 (2023): 1292–1306, 10.1111/all.15640. 36609802 PMC12727050

[55] S. Mijač , I. Banić , A.‐M. Genc , M. Lipej , and M. Turkalj , “The Effects of Environmental Exposure on Epigenetic Modifications in Allergic Diseases,” Medicina 60, no. 1 (2024): 110, 10.3390/medicina60010110. 38256371 PMC10820670

[56] L. Sun , J. Fu , S.‐H. Lin , et al., “Particulate Matter of 2.5 Μm or Less in Diameter Disturbs the Balance of TH17/Regulatory T Cells by Targeting Glutamate Oxaloacetate Transaminase 1 and Hypoxia‐Inducible Factor 1Α in an Asthma Model,” Journal of Allergy and Clinical Immunology 145, no. 1 (2020): 402–414, 10.1016/j.jaci.2019.10.008. 31647966

[57] R. Joglekar , C. Grenier , C. Hoyo , K. Hoffman , and S. K. Murphy , “Maternal Tobacco Smoke Exposure Is Associated With Increased DNA Methylation at Human Metastable Epialleles in Infant Cord Blood,” Environmental Epigenetics 8, no. 1 (2022 2022): dvac005, 10.1093/eep/dvac005. PMC8962709 35355955

[58] Y. S. Jung , J. Aguilera , A. Kaushik , et al., “Impact of Air Pollution Exposure on Cytokines and Histone Modification Profiles at Single‐Cell Levels During Pregnancy,” Science Advances 10, no. 48 (2024): eadp5227, 10.1126/sciadv.adp5227. 39612334 PMC11606498

[59] M. Khosrojerdi , F. J. Azad , Y. Yadegari , H. Ahanchian , and A. Azimian , “The Role of Micrornas in Atopic Dermatitis,” Noncoding RNA Research 9, no. 4 (2024): 1033–1039, 10.1016/j.ncrna.2024.05.012. PMC11254505 39022685

[60] J. Liu , M. Ballaney , U. Al‐alem , et al., “Combined Inhaled Diesel Exhaust Particles and Allergen Exposure Alter Methylation of T Helper Genes and Ige Production in Vivo,” Toxicological Sciences 102, no. 1 (2008): 76–81, 10.1093/toxsci/kfm290. 18042818 PMC2268643

[61] M. Xian , A. R. Maskey , D. Kopulos , and X.‐M. Li , “Advances of the Exposome at Individual Levels and Prevention in Atopic Dermatitis,” International Journal of Dermatology 64, no. 5 (2024): 794–808, 10.1111/ijd.17559. 39629600

[62] A. Belzer and E. R. Parker , “Climate Change, Skin Health, and Dermatologic Disease: A Guide for the Dermatologist,” American Journal of Clinical Dermatology 24, no. 4 (2023): 577–593, 10.1007/s40257-023-00770-y. 37336870

[63] Q. Guo , X. Xiong , F. Liang , et al., “The Interactive Effects Between Air Pollution and Meteorological Factors on the Hospital Outpatient Visits for Atopic Dermatitis in Beijing, China: A Time‐Series Analysis,” Journal of the European Academy of Dermatology and Venereology 33, no. 12 (2019): 2362–2370, 10.1111/jdv.15820. 31325384

[64] Z. Çelebi Sözener , E. R. Treffeisen , B. Özdel Öztürk , and L. C. Schneider , “Global Warming and Implications for Epithelial Barrier Disruption and Respiratory and Dermatologic Allergic Diseases,” Journal of Allergy and Clinical Immunology 152, no. 5 (2023): 1033–1046, 10.1016/j.jaci.2023.09.001. 37689250 PMC10864040

[65] J. Sato , M. Denda , S. Chang , P. M. Elias , and K. R. Feingold , “Abrupt Decreases in Environmental Humidity Induce Abnormalities in Permeability Barrier Homeostasis,” Journal of Investigative Dermatology 119, no. 4 (2002): 900–904, 10.1046/j.1523-1747.2002.00589.x. 12406336

[66] Y.‐M. Kim , J. Kim , K. Jung , S. Eo , and K. Ahn , “The Effects of Particulate Matter on Atopic Dermatitis Symptoms Are Influenced by Weather Type: Application of Spatial Synoptic Classification (SSC),” International Journal of Hygiene and Environmental Health 221, no. 5 (2018): 823–829, 10.1016/j.ijheh.2018.05.006. 29853291

[67] K. E. Burke and H. Wei , “Synergistic Damage by UVA Radiation and Pollutants,” Toxicology and Industrial Health 25, no. 4–5 (2009 2009): 219–224, 10.1177/0748233709106067. 19651790

[68] S. Song , K. Lee , Y.‐M. Lee , et al., “Acute Health Effects of Urban Fine and Ultrafine Particles on Children With Atopic Dermatitis,” Environmental Research 111, no. 3 (2011): 394–399, 10.1016/j.envres.2010.10.010. 21367405

[69] S.‐P. Wang , N. Stefanovic , R. L. Orfali , et al., “Impact of Climate Change on Atopic Dermatitis: A Review by the International Eczema Council,” Allergy 79, no. 6 (2024): 1455–1469, 10.1111/all.16007. 38265114

[70] L. Liu , C. Liu , R. Chen , et al., “Associations of Ambient Air Pollution and Daily Outpatient Visits for Pediatric Atopic Dermatitis in Shanghai, China,” Ecotoxicology and Environmental Safety 286 (2024): 117231, 10.1016/j.ecoenv.2024.117231. 39490101

[71] S. Ai , L. Liu , Y. Xue , X. Cheng , M. Li , and Q. Deng , “Prenatal Exposure to Air Pollutants Associated With Allergic Diseases in Children: Which Pollutant, When Exposure, and What Disease? A Systematic Review and Meta‐Analysis,” Clinical Reviews in Allergy and Immunology 66, no. 2 (2024): 149–163, 10.1007/s12016-024-08987-3. 38639856

[72] R. L. Miller , Y. Wang , J. Aalborg , et al., “Prenatal Exposure to Environmental Bisphenols Over Time and Their Association With Childhood Asthma, Allergic Rhinitis and Atopic Dermatitis in the ECHO Consortium,” Environment and Pollution 366 (2024): 125415, 10.1016/j.envpol.2024.125415. PMC12120670 39615574

[73] S. Lee , S.‐H. Ryu , W. J. Sul , S. Kim , D. Kim , and S. Seo , “Association of Exposure to Indoor Molds and Dampness With Allergic Diseases at Water‐Damaged Dwellings in Korea,” Scientific Reports 14, no. 1 (2024): 135, 10.1038/s41598-023-50226-w. 38167981 PMC10762174

[74] E. Lee , K. Y. Choi , M.‐J. Kang , et al., “Prenatal Mold Exposure Is Associated With Development of Atopic Dermatitis in Infants Through Allergic Inflammation,” Journal of Pediatrics 96, no. 1 (2020 2020): 125–131, 10.1016/j.jped.2018.07.012. PMC9432247 30243937

[75] Y. Ge , M. S. Nash , and A. K. Farraj , “Metabolomic Profiling Reveals Systemic Metabolic Disruptions Induced by Combined Exposure to Particulate Matter and Ozone,” Current Research in Toxicology 8 (2025): 100216, 10.1016/j.crtox.2025.100216. 39911777 PMC11795073

[76] S. Z. Celebi , B. Ozdel Ozturk , P. Cerci , et al., “Epithelial Barrier Hypothesis: Effect of the External Exposome on the Microbiome and Epithelial Barriers in Allergic Disease,” Allergy 77, no. 5 (2022): 1418–1449, 10.1111/all.15240. 35108405 PMC9306534

[77] W. Xiang and L. Chen , “In Light‐Sensitive Drug Delivery System Nanoparticles Mediate Oxidative Stress,” Journal of Translational Research 12, no. 5 (2020): 1469–1480, PMID: 32509156, PMCID: PMC7270018. PMC7270018 32509156

[78] S. Brown , S. J. Evans , M. J. Burgum , et al., “An In Vitro Model to Assess Early Immune Markers Following Co‐Exposure of Epithelial Cells to Carbon Black (Nano)Particles in the Presence of S. Aureus: A Role for Stressed Cells in Toxicological Testing,” Biomedicines 12, no. 1 (2024): 128, 10.3390/biomedicines12010128. 38255233 PMC10813740

[79] C. L. Chang , E. Berdyshev , E. Milanzi , et al., “Early‐Life Protein‐Bound Skin Ceramides Help Predict the Development of Atopic Dermatitis,” Journal of Allergy and Clinical Immunology 155, no. 3 (2025): 856–864, 10.1016/j.jaci.2024.10.041. 39945702

[80] E. Berdyshev , J. Kim , B. E. Kim , et al., “Stratum Corneum Lipid and Cytokine Biomarkers at Age 2 Months Predict the Future Onset of Atopic Dermatitis,” Journal of Allergy and Clinical Immunology 151, no. 5 (2023): 1307–1316, 10.1016/j.jaci.2023.02.013. 36828081

[81] R. Sanabria‐de la Torre , T. Montero‐Vílchez , J. García‐Gavín , and S. Arias‐Santiago , “Current Insights on Lipidomics in Dermatology: A Systematic Review,” Journal of Investigative Dermatology 145, no. 5 (2025): 1105–1116.e6, 10.1016/j.jid.2024.09.003. 39303909

[82] A. Hüls , C. Klümper , E. A. MacIntyre , et al., “Atopic Dermatitis: Interaction Between Genetic Variants of GSTP1, TNF, TLR2, and TLR4 and Air Pollution in Early Life,” Pediatric Allergy & Immunology 29, no. 6 (2018): 596–605, 10.1111/pai.12903. 29624745

[83] I. J. Wang , Y. L. Guo , T.‐J. Lin , P.‐C. Chen , and Y.‐N. Wu , “GSTM1, GSTP1, Prenatal Smoke Exposure, and Atopic Dermatitis,” Annals of Allergy, Asthma, & Immunology 105, no. 2 (2010): 124–129, 10.1016/j.anai.2010.04.017. 20674822

[84] C. Vogeley , S. Kress , D. Lang , et al., “A Gene Variant of AKR1C3 Contributes to Interindividual Susceptibilities to Atopic Dermatitis Triggered by Particulate Air Pollution,” Allergy 78, no. 5 (2023): 1372–1375, 10.1111/all.15622. 36524327 PMC10159942

[85] C. M. Khatib , A. W. Klein‐Petersen , A. T. M. Rønnstad , et al., “Increased Loss‐of‐Function Filaggrin Gene Mutation Prevalence in Atopic Dermatitis Patients Across Northern Latitudes Indicates Genetic Fitness: A Systematic Review and Meta‐Analysis,” Experimental Dermatology 33, no. 7 (2024): e15130, 10.1111/exd.15130. 38989976

[86] A. Simpson , S. L. John , F. Jury , et al., “Endotoxin Exposure, CD14, and Allergic Disease: An Interaction Between Genes and the Environment,” American Journal of Respiratory and Critical Care Medicine 174, no. 4 (2006): 386–392, 10.1164/rccm.200509-1380OC. 16614348

[87] D. Bučková , L. I. Hollá , V. Znojil , and A. Vašků , “Polymorphisms of the CD14 Gene and Atopic Phenotypes in Czech Patients With IgE‐Mediated Allergy,” Journal of Human Genetics 51, no. 11 (2006): 977–983, 10.1007/s10038-006-0050-0. 17003960

[88] A. Wollenberg , S. Barbarot , and A. Torrelo , “Basic Emollients for Xerosis Cutis in Atopic Dermatitis: A Review of Clinical Studies,” supplement, International Journal of Dermatology 64, no. S1 (2025): 13–28, 10.1111/ijd.17793. 40265493 PMC12124105

[89] P. V. Andrew , S. F. Williams , K. Brown , et al., “Topical Supplementation With Physiologic Lipids Rebalances the Stratum Corneum Ceramide Profile and Strengthens Skin Barrier Function in Adults Predisposed to Atopic Dermatitis,” British Journal of Dermatology (2025): ljaf200 Published Online May 23, 2025, 10.1093/bjd/ljaf200. 40408261

[90] P. M. Elias , “Optimizing Emollient Therapy for Skin Barrier Repair in Atopic Dermatitis,” Annals of Allergy, Asthma, & Immunology 128, no. 5 (2022): 505–511, 10.1016/j.anai.2022.01.012. PMC9979622 35065300

[91] P. V. Andrew , A. Pinnock , A. Poyner , et al., “Maintenance of an Acidic Skin Surface With a Novel Zinc Lactobionate Emollient Preparation Improves Skin Barrier Function in Patients with Atopic Dermatitis,” Dermatology and Therapy 14, no. 2 (2024): 391–408. 10.1007/s13555-023-01084-x. 38175365 PMC10891035

[92] H. M. U. L. Herath , M. J. Piao , K. A. Kang , P. D. S. M. Fernando , and J. W. Hyun , “Rosmarinic Acid Protects Skin Keratinocytes From Particulate Matter 2.5‐Induced Apoptosis,” International Journal of Medical Sciences 21, no. 4 (2024): 681–689, 10.7150/ijms.90814. 38464827 PMC10920844

[93] W. Preedalikit , C. Chittasupho , P. Leelapornpisid , N. Duangnin , and K. Kiattisin , “Potential of Coffee Cherry Pulp Extract Against Polycyclic Aromatic Hydrocarbons in Air Pollution Induced Inflammation and Oxidative Stress for Topical Applications,” International Journal of Molecular Sciences 25, no. 17 (2024): 9416, 10.3390/ijms25179416. 39273362 PMC11395326

[94] J.‐Y. Han , S.‐K. Kim , D.‐W. Lim , et al., “Anti‐Inflammatory Effect of Ethanol Extract From Hibiscus cannabinus L. Flower in Diesel Particulate Matter‐Stimulated Hacat Cells,” Nutrients 16, no. 22 (2024): 3805, 10.3390/nu16223805. 39599592 PMC11597620

[95] J. I. Silverberg , M. Boguniewicz , F. J. Quintana , et al., “Tapinarof Validates the Aryl Hydrocarbon Receptor as a Therapeutic Target: A Clinical Review,” Journal of Allergy and Clinical Immunology 154, no. 1 (2024): 1–10, 10.1016/j.jaci.2023.12.013. 38154665

[96] K. Damevska , V. Simeonovski , R. Darlenski , and S. Damevska , “How to Prevent Skin Damage From Air Pollution Part 2: Current Treatment Options,” Dermatologic Therapy 34, no. 6 (2021): e15132, 10.1111/dth.15132. 34528361

[97] A. Eftekhari , S. M. Dizaj , L. Chodari , et al., “The Promising Future of Nano‐Antioxidant Therapy Against Environmental Pollutants Induced‐Toxicities,” Biomedicine & Pharmacotherapy 103 (2018): 1018–1027, 10.1016/j.biopha.2018.04.126. 29710659

[98] X. Zhang , M. Yan , P. Chen , et al., “Emerging MOFs, COFs, and Their Derivatives for Energy and Environmental Applications,” Innovation; New York 6, no. 2 (2025): 100778, 10.1016/j.xinn.2024.100778. PMC11846040 39991481

[99] T. H. Vu , H.‐R. An , P. T. Nguyen , et al., “Large‐Sized and Highly Crystalline Ceria Nanorods With Abundant Ce^3+^ Species Achieve Efficient Intracellular ROS Scavenging,” Nanoscale Horiz 10, no. 4 (2025): 791–802, 10.1039/d4nh00639a. 39949300

[100] D. Y. Lin , S. T. Waller , and M. Y. Lin , “A Review of Urban Planning Approaches to Reduce Air Pollution Exposures,” Current Environmental Health Reports 11, no. 4 (2024): 557–566, 10.1007/s40572-024-00459-2. 39198370

[101] N. Rawat and P. Kumar , “Interventions for Improving Indoor and Outdoor Air Quality in and Around Schools,” Science of the Total Environment 858, no. Pt 2 (2023): 159813, 10.1016/j.scitotenv.2022.159813. 36411671

[102] M. Sharma and S. Dasappa , “Emission Reduction Potentials of Improved Cookstoves and Their Issues in Adoption: An Indian Outlook,” Journal of Environmental Management 204, no. Pt 1 (2017): 442–453, 10.1016/j.jenvman.2017.09.018. 28917179

[103] L. Buckley , C. A. Arter , M. D. Willis , et al., “A Comparison of Population‐Level Exposure and Equity Tradeoffs Across Strategies to Reduce Fine Particulate Matter Emissions From Transportation Sources in the Northeastern US,” Environmental Research 262, no. Pt 1 (2024): 119791, 10.1016/j.envres.2024.119791. 39151555

[104] Z. Shao , X. Zheng , J. Zhao , and Y. Liu , “Evaluating the Health Impact of Air Pollution Control Strategies and Synergies Among PM2.5 and O3 Pollution in Beijing‐Tianjin‐Hebei Region, China,” Environmental Research 274 (2025): 121348, 10.1016/j.envres.2025.121348. 40058552

[105] T. Mora , I. Sánchez‐Collado , R. Muñoz‐Cano , et al., “Prevalence and Coexistence of Type 2 Inflammatory Diseases,” Clinical and Translational Allergy 14, no. 6 (2024): e12376, 10.1002/clt2.12376. 38898824 PMC11187401

[106] P. Losol , M. Wolska , T. P. Wypych , L. Yao , L. O’Mahony , and M. Sokolowska , “A Cross Talk Between Microbial Metabolites and Host Immunity: Its Relevance for Allergic Diseases,” Clinical and Translational Allergy 14, no. 2 (2024): e12339, 10.1002/clt2.12339. 38342758 PMC10859320

